# Pulmonary ultrasound and pulse oximetry versus chest radiography and arterial blood gas analysis for the diagnosis of acute respiratory distress syndrome: a pilot study

**DOI:** 10.1186/s13054-015-0995-5

**Published:** 2015-07-21

**Authors:** Cameron M. Bass, Dana R. Sajed, Adeyinka A. Adedipe, T. Eoin West

**Affiliations:** Department of Medicine, University of Washington School of Medicine, Seattle, WA USA; International Respiratory and Severe Illness Center, University of Washington School of Medicine, Seattle, WA USA

## Abstract

**Introduction:**

In low-resource settings it is not always possible to acquire the information required to diagnose acute respiratory distress syndrome (ARDS). Ultrasound and pulse oximetry, however, may be available in these settings. This study was designed to test whether pulmonary ultrasound and pulse oximetry could be used in place of traditional radiographic and oxygenation evaluation for ARDS.

**Methods:**

This study was a prospective, single-center study in the ICU of Harborview Medical Center, a referral hospital in Seattle, Washington, USA. Bedside pulmonary ultrasound was performed on ICU patients receiving invasive mechanical ventilation. Pulse oximetric oxygen saturation (SpO_2_), partial pressure of oxygen (PaO_2_), fraction of inspired oxygen (FiO_2_), provider diagnoses, and chest radiograph closest to time of ultrasound were recorded or interpreted.

**Results:**

One hundred and twenty three ultrasound assessments were performed on 77 consecutively enrolled patients with respiratory failure. Oxygenation and radiographic criteria for ARDS were met in 35 assessments. Where SpO_2_ ≤ 97 %, the Spearman rank correlation coefficient between SpO_2_/FiO_2_ and PaO_2_/FiO_2_ was 0.83, *p* < 0.0001. The sensitivity and specificity of the previously reported threshold of SpO_2_/FiO_2_ ≤ 315 for PaO_2_/FiO_2_ ≤ 300 was 83 % (95 % confidence interval (CI) 68–93), and 50 % (95 % CI 1–99), respectively. Sensitivity and specificity of SpO_2_/FiO_2_ ≤ 235 for PaO_2_/FiO_2_ ≤ 200 was 70 % (95 % CI 47–87), and 90 % (95 % CI 68–99), respectively. For pulmonary ultrasound assessments interpreted by the study physician, the sensitivity and specificity of ultrasound interstitial syndrome bilaterally and involving at least three lung fields were 80 % (95 % CI 63–92) and 62 % (95 % CI 49–74) for radiographic criteria for ARDS. Combining SpO_2_/FiO_2_ with ultrasound to determine oxygenation and radiographic criteria for ARDS, the sensitivity was 83 % (95 % CI 52–98) and specificity was 62 % (95 % CI 38–82). For moderate–severe ARDS criteria (PaO_2_/FiO_2_ ≤ 200), sensitivity was 64 % (95 % CI 31–89) and specificity was 86 % (95 % CI 65–97). Excluding repeat assessments and independent interpretation of ultrasound images did not significantly alter the sensitivity measures.

**Conclusions:**

Pulse oximetry and pulmonary ultrasound may be useful tools to screen for, or rule out, impaired oxygenation or lung abnormalities consistent with ARDS in under-resourced settings where arterial blood gas testing and chest radiography are not readily available.

**Electronic supplementary material:**

The online version of this article (doi:10.1186/s13054-015-0995-5) contains supplementary material, which is available to authorized users.

## Introduction

Acute respiratory distress syndrome (ARDS) is a common cause of mortality in the ICU [1]. The diagnosis of ARDS is established using the new Berlin criteria which consists of four elements: 1) onset within a week of a known clinical insult or new respiratory symptoms, 2) bilateral opacities on chest radiograph or computed tomography scan, 3) respiratory failure not fully explained by cardiac failure or fluid overload, and 4) impaired oxygenation defined as partial pressure of oxygen (PaO_2_)/fraction of inspired oxygen (FiO_2_) ≤300 on positive end-expiratory pressure (PEEP) or continuous positive airway pressure (CPAP) ≥5 cmH_2_O. Diagnosis of ARDS requires an arterial blood gas (ABG) test and chest radiography or computed tomography in the appropriate clinical scenario.

In much of the world where medical resources are limited, blood gas analysis and imaging technologies may not be available, impairing the ability to make the diagnosis of ARDS. In one study, half of all patients who clinically had ARDS in a hospital in Rwanda had a chest radiograph available for review [2]. However, both pulse oximetry and ultrasound are becoming increasingly accessible worldwide [3, 4]. The pulse oximetric saturation to inspired oxygen ratio (SpO_2_/FiO_2_) has been correlated with the PaO_2_/FiO_2_ ratio in ARDS [5, 6]. Pulmonary ultrasound is a rapidly developing technology in which the diagnosis of lung disease is being explored in diverse settings, and new diagnostic criteria are being developed for multiple pulmonary processes [7]. Some of the first patterns of pulmonary ultrasound to be recognized were the distinct “A line” and “B line” artifacts [8]. The “A-line” pattern, characterized by horizontal reflection artifacts of the pleural line deep into the lung, is seen with alveoli that are physiologically filled with air. The “B-line” pattern, characterized by the presence of three or more vertical artifacts obliterating any A-lines, correlates with the ultrasound interstitial syndrome (UIS) [9]. The presence of UIS diffusely on ultrasound is considered consistent with either cardiogenic pulmonary edema or ARDS [10–12]. The A line and B line patterns have proven to be easily distinguished by a bedside clinician after relatively brief teaching [13, 14]. Therefore, it is conceivable that the diagnosis of ARDS could be made using pulse oximetry and pulmonary ultrasound at the point of care.

We hypothesized that data derived from pulse oximetry and bedside pulmonary ultrasound could be used in lieu of ABG and chest radiography to meet oxygenation and radiographic criteria for ARDS. We designed a prospective study in patients with respiratory failure in the ICU to test this hypothesis.

## Methods

### Study procedures

The study was conducted in the ICUs at Harborview Medical Center, Seattle, Washington, USA. The study physician received 4 h of hands-on pulmonary ultrasound training from an ultrasound fellowship-trained emergency medicine attending physician. Training included ultrasound scanning and discussions at the bedside of ten patients with different lung pathologies and a brief literature review.

**Fig. 1:**
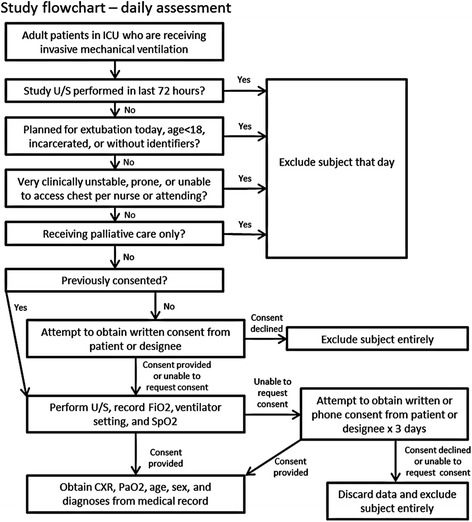
Study flow chart. *CXR* chest x-ray, *FiO2* fraction of inspired oxygen, *PaO2* partial pressure of oxygen, *SpO2*, pulse oximetric oxygen saturation, *U/S* ultrasound

From 4 July to 22 August 2013, mechanically ventilated patients in the ICUs were identified by the study physician early each morning using a Quality Safety Dashboard, Monday through Friday. Patients were screened for study eligibility based on inclusion and exclusion criteria (Fig. 1). Although the study design initially included patients on high levels of supplemental oxygen via high flow nasal cannula or face mask, the revised Berlin definition of ARDS published in 2012 required administration of CPAP or PEEP, so eligibility was restricted to patients receiving mechanical ventilation. No attempts were made to determine the etiology or management of respiratory failure before enrollment. Contraindications included burns over the chest, flail chest, active hemodynamic instability or declination by the patient’s nurse, receiving palliative care, age less than 18 years, incarceration, prone positioning, planned extubation the morning of study, or lack of identifying personal information.

**Fig. 2:**
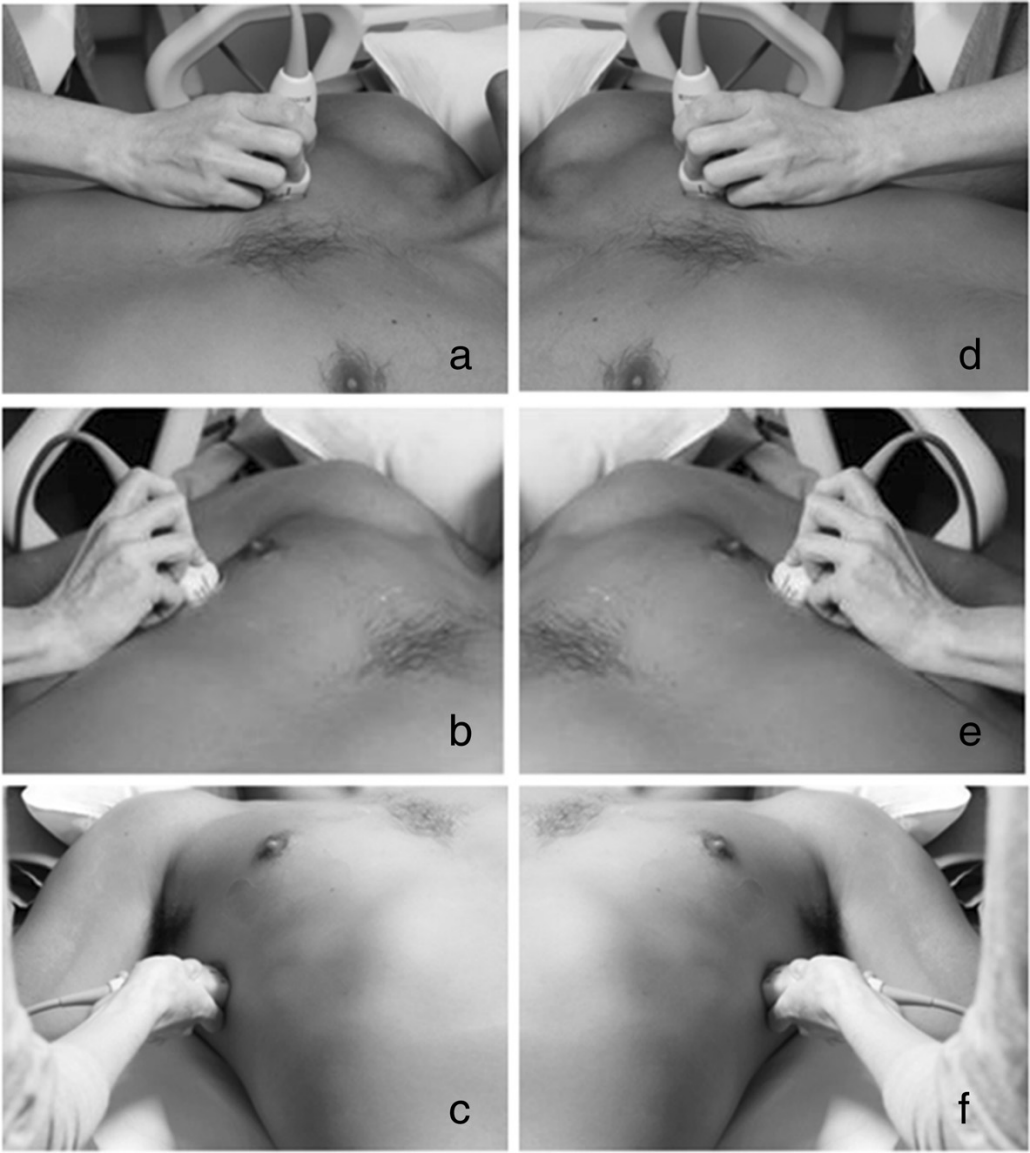
Placement of the ultrasound probe at six locations on the chest. **a** zone 1 is 2 cm below the anterior mid-clavicular line on the right side of the chest; **b** zone 2 is 4 cm inferior and 4 cm lateral to zone 1; **c** zone 3 is 2 cm inferior to zone 2 along the mid-axillary line. **d**–**f** The identical positions on the left side of the chest

**Fig. 3:**
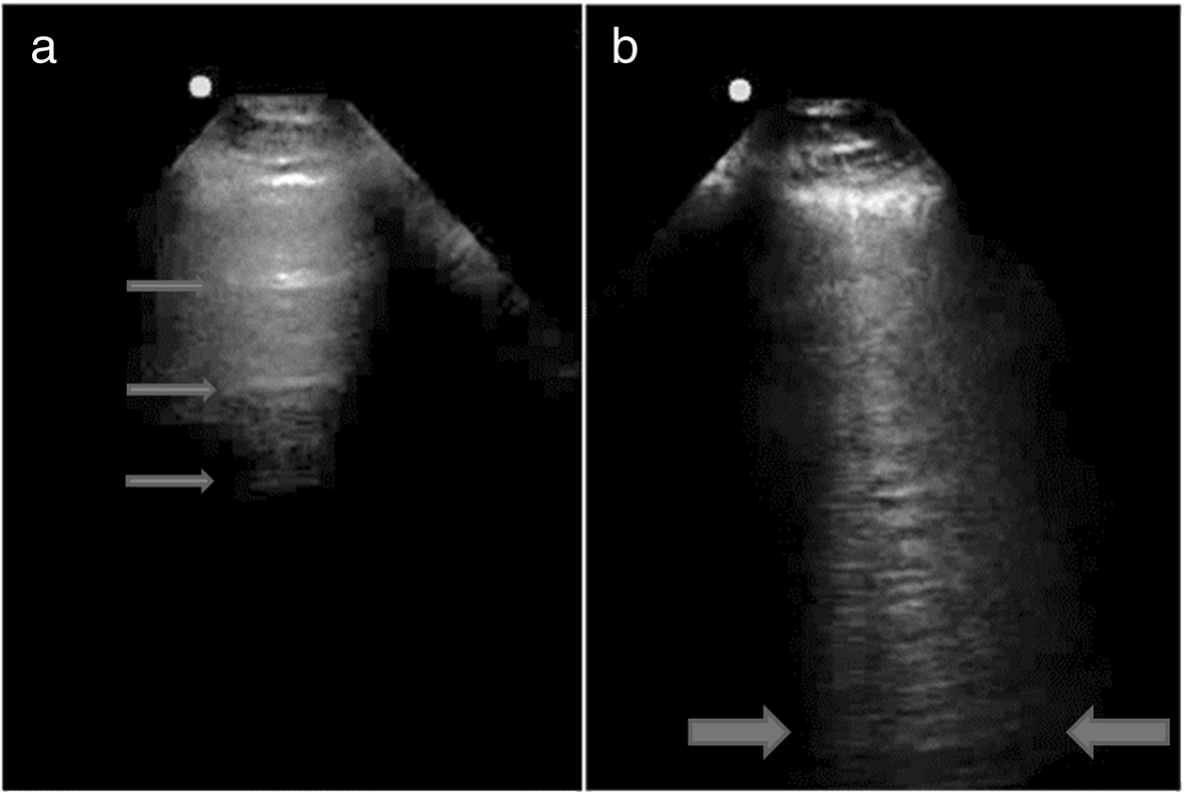
**a** “A lines”: distinct horizontal reflections in a patient with normal lungs (*arrows*). **b** “B lines”: three vertical lines in a single frame extending from the pleura to the bottom of the screen in a patient with ultrasound interstitial syndrome (between *arrows*)

For patients who met eligibility criteria the study physician performed an ultrasound evaluation prior to reviewing the patient’s chart or other imaging. A Sonosite S-ICU ultrasound machine (SonoSite, Inc., Bothell, Washington) with a p21x 5–1 Mhz phased array probe was used to capture 6-second video imaging of the lung at three sites on each side of the chest (Fig. 2), following a modification of a previously described protocol [10, 15, 16]. Specifically, the six “BLUE” points were evaluated for the presence of B lines (Fig. 3). In order to mimic rapid evaluation by a single clinician in austere conditions, patients were not repositioned for the purpose of the scan and assistance from additional staff members was not requested. No scan was permitted to take more than 5 min, including start up time for the machine and recording time for the videos. The FiO_2_ and SpO_2_ at the time of the ultrasound were recorded. This procedure was performed as close to 06:30 am as possible to reduce the duration between ultrasound and early morning chest radiographs and ABG measurements performed in the ICUs. Subsequently, the chest radiograph and ABG performed closest to the time of the ultrasound were abstracted from the medical record. The FiO_2_ and SpO_2_ recorded by transcutaneous probe at the time of the ABG were also recorded. The ICU teams clinical note for the day of the study evaluation was reviewed to capture active clinical diagnoses. If patients continued to meet eligibility criteria, repeat study assessments were performed every 3 days.

### Definitions

For each study assessment, the ABG and concurrent FiO_2_ data was used to calculate a PaO_2_/FiO_2_ value. Chest radiographs were independently reviewed by a board-certified attending chest radiologist to determine if they met radiographic criteria for ARDS, namely bilateral opacities not fully explained by effusions, lobar/lung collapse, or nodules [17]. Computerized tomography scans of the chest were not available for all patients, so this modality was not incorporated into the study design. The radiologist was blinded to all other clinical information. Study assessments with PaO_2_/FiO_2_ ≤ 300 and a chest radiograph interpreted as consistent with ARDS were deemed to have met oxygenation and radiographic criteria for ARDS [17]. PaO_2_/FiO_2_ ≤ 200 was considered at least moderately impaired oxygenation.

For each study assessment the SpO_2_/FiO_2_ ratio was calculated using SpO_2_ and FiO_2_ recorded at the time of the ABG. Based on the relationship between SpO_2_/FiO_2_ and PaO_2_/FiO_2_ in ARDS patients derived by Rice et al. [5], SpO_2_/FiO_2_ ≤ 315 was considered impaired oxygenation and SpO_2_/FiO_2_ ≤ 235 at least moderately impaired oxygenation. UIS was defined as at least three B lines in a single frozen frame in one or more lung fields. Ultrasound imaging consistent with ARDS was determined by the presence of UIS bilaterally [7, 10, 12, 15]. Images that were difficult to interpret due to subcutaneous emphysema, obesity, consolidations, effusions, image quality or positioning were noted, but were still designated as consistent with UIS or not. All ultrasound video clips were batch reviewed and classified by the study physician and independently reviewed and classified by an ultrasound-trained attending physician in a blinded manner after the completion of patient enrollment.

### Statistics

Continuous data were displayed as mean ± standard deviation if normally distributed or median and interquartile range (IQR) if non-normally distributed. Correlation between continuous variables was determined using Spearman’s rank correlation coefficient. Diagnostic accuracy measures of sensitivity and specificity were calculated with 95 % exact binomial confidence intervals (CIs). Interobserver agreement was determined using the kappa coefficient. Nonparametric receiver operating curve analysis was used to determine area under the curve (AUC). We treated each assessment as independent in our primary analysis based on frequent changes in individual patients’ clinical status over 3-day intervals, but we also performed a sensitivity analysis restricted to initial assessments. STATA v11.2 (College Station, TX, USA) was used for statistical analyses.

### Human subjects

This study was approved by the University of Washington Institutional Review Board. Informed consent was obtained from patients or their designee.

## Results

### Patients and study assessments

One hundred and twenty three study assessments were conducted on 77 patients, all of whom were mechanically ventilated and on PEEP of at least 5 cmH_2_O. The characteristics of the study patients and assessments are given in Table 1. The median age of patients was 56 years (IQR 41–67); 52 (68 %) were male, and 24 patients underwent more than one assessment. Eight (33 %) were reclassified with respect to oxygenation criteria for ARDS and 9 (38 %) were reclassified with respect to radiographic criteria for ARDS over serial assessments. These changes included both reclassification as meeting criteria and reclassification as no longer meeting criteria. As patients were assessed no more frequently than every 3 days and because their clinical status often changed significantly in that time period, we chose assessments as our unit of analysis, treating them as independent. The most common diagnoses at the time of assessments were sepsis (n = 36, 29 %) and trauma (n = 31, 25 %).

**Table 1:** Baseline characteristics of subjects and study assessments

Characteristic	Number	Percent
Patients	77	
Male	52	68
Age, median (IQR)	56 (41–67)	
Number undergoing 2 assessments	13	17
Number undergoing 3 assessments	3	4
Number undergoing ≥4 assessments	8	10
Assessments	123	
Site:		
MICU	45	37
SICU	49	40
NICU	29	24
Diagnosis:		
Sepsis	36	29
Trauma	31	25
Postsurgery	24	20
CVA	21	17
Cardiogenic pulmonary edema	21	17
Pneumonia	20	16
PEA or VF arrest	13	11
PE	10	8
ARDS	10	8
Overdose	9	7
Seizure	7	6
Pancreatitis	7	6
COPD	6	5
FiO_2_ at time of ABG, median (IQR)	0.40 (0.30–0.50)	
PaO_2_/FiO_2_, median (IQR)	250 (180–337)	
SpO_2_ % at time of ABG, median (IQR)	99 (97–100)	
Bilateral opacities on CXR	42	34

Patients may have more than one diagnosis at the time of assessment. *ABG* arterial blood gas, *ARDS* acute respiratory distress syndrome, *COPD* chronic obstructive pulmonary disease, *CVA* Cerebrovascular Accident, *CXR* chest x-ray, *IQR* interquartile range, *MICU* Medical Intensive Care Unit, *NICU* Neurological Intensive Care Unit, *PE* Pulmonary Embolism, *PEA* Pulseless Electrical Activity, *SICU* Surgical Intensive Care Unit, *SpO2* pulse oximetric oxygen saturation, *VF* Ventricular Fibrillation

### Relationship between SpO_2_/FiO_2_ and PaO_2_/FiO_2_

**Fig. 4:**
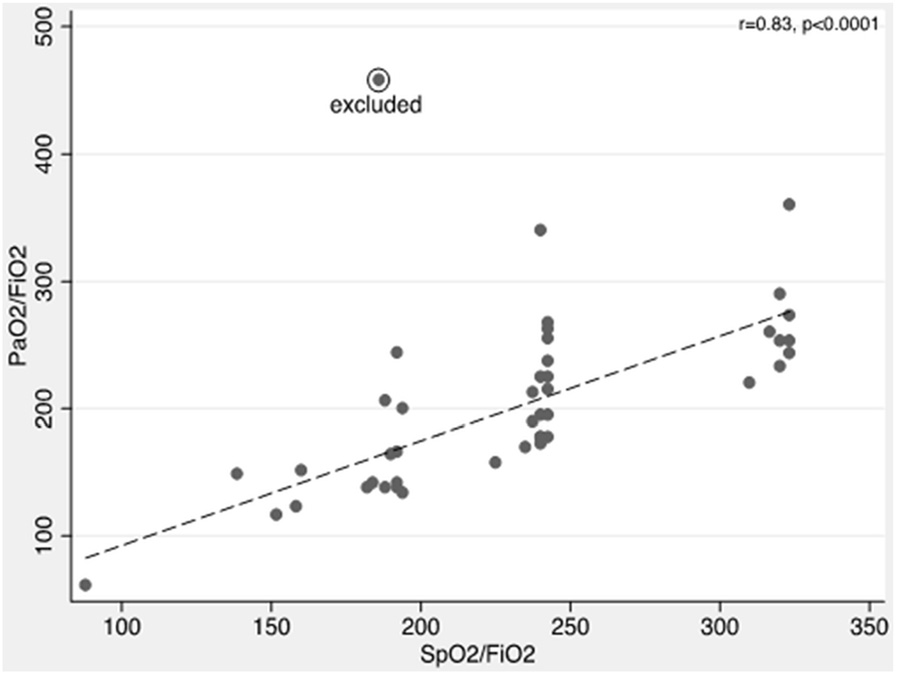
Correlation between SpO_2_/FiO_2_ and PaO_2_/FiO_2_ when SpO_2_ ≤ 97 %. *FiO2* fraction of inspired oxygen, *PaO2* partial pressure of oxygen, *SpO2* pulse oximetric oxygen saturation

At the time of ultrasound the median FiO_2_ was 0.40 (IQR 0.30–0.40) and the median SpO_2_ was 98 % (IQR 96–100). At the time of ABG, the median FiO_2_ was 0.40 (IQR 0.30–0.50), median SpO_2_ was 99 % (IQR 97–100), and median PaO_2_ was 100 (IQR 78–122). The SpO_2_ at the time of ABG was used for all further analyses. The relationship between SpO_2_/FiO_2_ and PaO_2_/FiO_2_ derived by Rice et al. in a cohort of ARDS patients indicated that SpO_2_/FiO_2_ threshold values of 315 and 235 corresponded to PaO_2_/FiO_2_ of 300 and 200, respectively, when SpO_2_ ≤ 97 % [5]. To validate this relationship in our cohort of mechanically ventilated patients, we restricted our analysis to the 44 observations where SpO_2_ ≤ 97 % at the time of ABG. In this subset, the median PaO_2_/FiO_2_ was 198 (IQR 155–249) and the median SpO_2_/FiO_2_ was 240 (IQR 191–243). The Spearman rank correlation coefficient between PaO_2_/FiO_2_ and SpO_2_/FiO_2_ was 0.74 (*p* < 0.0001). We identified one outlier that was characterized by marked discordance between PaO_2_ and SpO_2_ due to rapidly dynamic changes in oxygenation at the time of the study. Excluding this observation, the correlation between PaO_2_/FiO_2_ and SpO_2_/FiO_2_ increased to 0.83 (*p* < 0.0001) (Fig. 4). We performed receiver operating curve analysis to further evaluate the SpO_2_/FiO_2_ ratio. SpO_2_/FiO_2_ ratio had modest ability to discriminate PaO_2_/FiO_2_ ≤ 300, based on an AUC value of 0.76 (95 % CI 0.34–1.00) (Additional file 1: Figure S1A). The discriminatory ability for SpO_2_/FiO_2_ in classifying PaO_2_/FiO_2_ ≤ 200, however, was considerably better, with an AUC of 0.89 (95 % CI 0.80–0.99) (Additional file 1: Figure S1B). The sensitivity of SpO_2_/FiO_2_ ≤ 315 for PaO_2_/FiO_2_ ≤ 300 was 83 % (95 % CI 68–93), and the specificity was 50 % (95 % CI 1–99) (Table 2). The sensitivity of SpO_2_/FiO_2_ ≤ 235 for PaO_2_/FiO_2_ ≤ 200 was 70 % (95 % CI 47–87), and the specificity was 90 % (95 % CI 68–99).

**Table 2:** Performance of SpO_2_/FiO_2_ as a marker of PaO_2_/FiO_2_ when SpO_2_ ≤ 97 %

Number of observations (n = 43)				Test characteristic		95 % CI
		PaO_2_/FiO_2_				
		≤300	>300	Sensitivity	83 %	68–93
SpO_2_/FiO_2_	≤315	34	1	Specificity	50 %	1–99
	>315	7	1	PPV	97 %	85–100
				NPV	13 %	0–53
		PaO_2_/FiO_2_				
		≤200	>200	Sensitivity	70 %	47–87
SpO_2_/FiO_2_	≤235	16	2	Specificity	90 %	68–99
	>235	7	18	PPV	89 %	65–99
				NPV	72 %	51–88

*CI* confidence interval, *FiO*_*2*_ fraction of inspired oxygen, *NPV* negative predictive value, *PaO*_*2*_ partial pressure of oxygen, *PPV* positive predictive value, *SpO*_*2*_ pulse oximetric oxygen saturation

### UIS as a marker for radiographic opacities consistent with ARDS

**Fig. 5:**
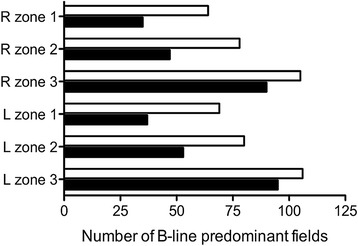
Distribution of B line-predominant lung fields. *Black bars* indicate reads by the study physician; *white bars* indicate reads by the independent physician. For right (*R*) and left (*L*), zones correspond to locations shown in Fig. 2

Of the 738 lung fields evaluated by ultrasound (six fields for 123 assessments), 357 (48 %) demonstrated B line predominance as interpreted by the study physician. B lines were more common in posterior lung fields but were distributed equally on the left and on the right (Fig. 5). One hundred and one ultrasound assessments were conducted within 8 h of a chest radiograph. We used this subset of assessments to evaluate optimal thresholds of UIS for determination of radiographic criteria of ARDS as various thresholds have been reported [7, 10–12, 15, 18]. In 35 (35 %) assessments, bilateral opacities consistent with ARDS were apparent on chest radiograph. The sensitivity and specificity of UIS in at least one lung field bilaterally (UIS-2) for radiographic ARDS were 86 % (95 % CI 70–95) and 38 % (95 % CI 26–51) (Table 3). The sensitivity and specificity of UIS in at least one field bilaterally and involving a minimum of three lung fields (UIS-3) were 80 % (95 % CI 63–92) and 62 % (95 % CI 49–74). The sensitivity and specificity of UIS in at least two lung fields bilaterally (UIS-4) were 60 % (95 % CI 42–76) and 77 % (95 % CI 65–87). By receiver operating curve analysis, the ability of UIS pattern to discriminate radiographic ARDS was fair (AUC 0.73, 95 % CI 0.63–0.83) (Additional file 2: Figure S2).

**Table 3:** Performance of ultrasound diagnosis of ultrasound interstitial syndrome (UIS) as a marker of bilateral pulmonary opacities consistent with acute respiratory distress syndrome

UIS threshold		Number of observations (n = 101)			Test characteristic		95 % CI
1 lung field bilaterally			Bilateral opacities on chest radiograph				
			Present	Absent	Sensitivity	86 %	70–95
	UIS	Present	30	41	Specificity	38 %	26–51
		Absent	5	25	PPV	42 %	31–55
					NPV	83 %	65–94
Bilateral; 3 lung fields minimum			Bilateral opacities on chest radiograph				
			Present	Absent	Sensitivity	80 %	63–92
	UIS	Present	28	25	Specificity	62 %	49–74
		Absent	7	41	PPV	53 %	39–67
					NPV	85 %	72–94
2 lung fields bilaterally			Bilateral opacities on chest radiograph				
			Present	Absent	Sensitivity	60 %	42–76
	UIS	Present	21	15	Specificity	77 %	65–87
		Absent	14	51	PPV	58 %	41–75
					NPV	79 %	67–88

*CI* confidence interval, *NPV* negative predictive value, *PPV* positive predictive value

### Interobserver reliability of UIS interpretation

To test interobserver reliability of the ultrasound interpretations, all ultrasound videos were independently reviewed by an ultrasound-trained attending physician who had not participated in training or data collection and who was blinded to the clinical scenario. The kappa coefficient between the two interpreters for designating B lines present in a lung field was 0.57 (CI 0.40–0.73), consistent with moderate agreement. In comparison to the study physician, the independent physician classified a higher proportion of lung fields as B line-predominant but the relative distribution of B lines in the left lung compared to right lung, and throughout the upper, middle, and lower lung zones was comparable (Fig. 5). We evaluated the interobserver reliability for different UIS thresholds. For UIS-2, the kappa coefficient was 0.44 (CI 0.05–0.83); for UIS-3, the kappa coefficient was 0.45 (CI 0.09–0.81); and for UIS-4, the kappa coefficient was 0.47 (CI 0.07–0.87). Using the independent physician’s classifications, sensitivity and specificity of UIS-2 for radiographic criteria of ARDS were 89 % (95 % CI 73–97) and 15 % (95 % CI 8–26), respectively; 89 % (95 % CI 73–97) and 29 % (95 % CI 18–41) for UIS-3; and 83 % (95 % CI 66–93) and 46 % (95 % CI 33–58) for UIS-4 (Table 3).

### SpO_2_/FiO_2_ and UIS as marker for oxygenation and radiographic criteria for ARDS

We next evaluated the combination of pulse oximetry measurements and pulmonary ultrasound assessments as markers for the coexistence of oxygenation and radiographic criteria for ARDS in our cohort. We combined SpO_2_/FiO_2_ ≤ 315 and ultrasound demonstrating UIS-3 in the subset of 33 observations with SpO_2_ ≤ 97 %, a chest radiograph within 8 h of ultrasound, and ABG within 24 h of ultrasound (Table 4). The sensitivity of the combination of pulse oximetry and ultrasound determinations of oxygenation and radiographic ARDS criteria was 83 % (95 % CI 52–98) and specificity was 62 % (95 % CI 38–82). The positive predictive value was 56 % (95 % CI 31–79) and the negative predictive value was 87 % (95 % CI 60–98). We repeated this analysis using a threshold SpO_2_/FiO_2_ ≤ 235, restricting to cases of at least moderate ARDS (PaO_2_/FiO_2_ ≤ 200) and found that the sensitivity was 64 % (95 % CI 31–89) and specificity was 86 % (95 % CI 65–97). The positive predictive value was 70 % (95 % CI 35–93) and the negative predictive value was 83 % (95 % CI 61–95).

**Table 4:** Performance of SpO_2_/FiO_2_ and ultrasound interstitial syndrome (UIS) as a marker for with acute respiratory distress syndrome (ARDS) criteria when SpO_2_ ≤ 97 %

		Number of observations (n = 33)		Test characteristic		95 % CI
Study physician		Oxygenation and radiographic criteria for ARDS				
		Present	Absent	Sensitivity	83 %	52–98
SpO_2_/FiO_2_ ≤ 315 and UIS	Present	10	8	Specificity	62 %	38–82
	Absent	2	13	PPV	56 %	31–79
				NPV	87 %	60–98
		Oxygenation and radiographic criteria for moderate–severe ARDS				
		Present	Absent	Sensitivity	64 %	31–89
SpO_2_/FiO_2_ ≤ 235 and UIS	Present	7	3	Specificity	86 %	65–97
	Absent	4	19	PPV	70 %	35–93
				NPV	83 %	61–95
Independent physician		Oxygenation and radiographic criteria for ARDS				
		Present	Absent	Sensitivity	91 %	62–100
SpO_2_/FiO_2_ ≤ 315 and UIS	Present	11	11	Specificity	48 %	26–70
	Absent	1	10	PPV	50 %	28–72
				NPV	91 %	59–100
		Oxygenation and radiographic criteria for moderate–severe ARDS				
		Present	Absent	Sensitivity	73 %	39–94
SpO_2_/FiO_2_ ≤ 235 and UIS	Present	8	5	Specificity	77 %	55–92
	Absent	3	17	PPV	62 %	32–86
				NPV	85 %	62–97

UIS defined as 3 or more B lines bilaterally and involving a minimum of three lung fields. *CI* confidence interval, *FiO*_*2*_ fraction of inspired oxygen, *NPV* negative predictive value, *PaO*_*2*_ partial pressure of oxygen, *PPV* positive predictive value, *SpO*_*2*_ pulse oximetric oxygen saturation

We considered the ultrasound interpretations of the independent physician and repeated these analyses. The sensitivity of the combination of pulse oximetry (SpO_2_/FiO_2_ ≤ 315) and ultrasound (UIS-3) for oxygenation and radiographic ARDS criteria was 91 % (95 % CI 62–100) and specificity was 48 % (95 % CI 26–70) (Table 4). The sensitivity of the combination of pulse oximetry (SpO_2_/FiO_2_ ≤ 235) and ultrasound (UIS-3) for oxygenation and radiographic criteria of severe ARDS was 73 % (95 % CI 39–94) and specificity was 77 % (95 % CI 55–92).

### Sensitivity to repeat assessments

To determine whether our treatment of each assessment as independent altered our findings, we performed sensitivity analyses restricted to the first assessment for each of the 77 patients. We did not find any significant differences in the relationships between SpO_2_/FiO_2_ and PaO_2_/FiO_2_. Diagnostic accuracy of UIS as a marker for radiographic opacities consistent with ARDS was also similar: the sensitivity and specificity of UIS-3 were 80 % (95 % CI 56–94) and 72 % (95 % CI 57–84). The sensitivity of the combination of pulse oximetry and ultrasound determinations of oxygenation and radiographic ARDS criteria (SpO_2_/FiO_2_ ≤ 315 and UIS-3) was 88 % (95 % CI 47–100) and specificity was 69 % (95 % CI 39–91).

## Discussion

The purpose of this pilot study was to assess the performance of rapid assessment with bedside pulmonary ultrasound and use of pulse oximetry as alternatives to chest radiograph and ABG in the diagnosis of ARDS. The results of this study showed that, in mechanically ventilated ICU patients, SpO_2_/FiO_2_ and PaO_2_/FiO_2_ are highly correlated, that SpO_2_/FiO_2_ ≤ 315 is quite sensitive for PaO_2_/FiO_2_ ≤ 300, and that SpO_2_/FiO_2_ ≤ 235 is highly specific for PaO_2_/FiO_2_ ≤ 200. Our study confirms reasonable sensitivity of simplified six-point lung ultrasound in identifying patients with bilateral pulmonary opacities consistent with ARDS on chest radiograph using a threshold of bilateral UIS involving at least three lung fields in total, although specificity was lower. Finally, our data on a relatively small number of patients indicate that the combination of SpO_2_/FiO_2_ ≤ 315 and bilateral/3 field UIS on ultrasound is sensitive for the classification of traditional oxygenation and radiographic criteria for ARDS in mechanically ventilated patients; in contrast, the combination of SpO_2_/FiO_2_ ≤ 235 and bilateral/3 field UIS on ultrasound is specific for moderate–severe ARDS.

Overall, we found that the SpO_2_/FiO_2_ cutoffs established by Rice et al. were less predictive of the PaO_2_/FiO_2_ than originally described [5]. Rice et al. found higher sensitivity in their study, with SpO_2_/FiO_2_ ≤ 235 resulting in 85 % sensitivity with 85 % specificity for PaO_2_/FiO_2_ ≤ 200, and 91 % sensitivity with 56 % specificity of SpO_2_/FiO_2_ ≤ 315 to predict PaO_2_/FiO_2_ ≤ 300. Several explanations may account for this. First, the use of the SpO_2_/FiO_2_ ratio is limited by flattening of the oxyhemoglobin dissociation curve at high SpO_2_; this effect is exacerbated when FiO_2_ is not maximally reduced. Many patients in our study were administered a “minimum” FiO_2_ of 0.4. Rice et al. report that their studies targeted SpO_2_ values between 92 and 94 %, whereas very few of our subjects had SpO_2_ < 97 %. Second, Rice et al. analyzed 1,076 patients enrolled in ARDS studies; this contrasts markedly with our smaller, relatively unfiltered cohort of 77 mechanically ventilated patients. Third, our study is a single-center observational study, in contrast to the multicenter interventional ARDSNet studies. Future studies refining test characteristics of new ARDS criteria should determine SpO_2_/FiO_2_ at the lowest possible FiO_2_, which would require cooperation with respiratory therapists and nursing staff.

The sensitivity and specificity of ultrasound assessments of UIS for radiographic criteria of ARDS in our study were also less than was predicted based on prior studies. There are several possible reasons for this. The original diagnostic algorithm for the BLUE protocol includes an assessment of the lung sliding by ultrasound [10]. This specific criterion was removed from our study, as in trauma patients with chest injuries one might expect a loss of lung sliding without ruling out ARDS or cardiogenic pulmonary edema. An additional challenge in the trauma patient is the evaluation in the setting of significant subcutaneous emphysema. In this setting ultrasound images are difficult to interpret, and may provide false reassurance to novice sonographers simply counting B lines or looking for hepatization. A phased-array probe was used in this study instead of the micro convex probe used in many other studies. This was chosen based on an assessment that the two probes most likely to be found in a resource limited setting were a linear probe (for superficial assessments and procedures) and a phased-array probe (for cardiac, intra-abdominal, and obstetric assessment). In addition, unlike other studies [10, 11, 18], our study used only chest radiographs and not chest computed tomography imaging to determine radiographic criteria for ARDS.

About a third of ultrasound assessments we performed had at least one lung field for which the imaging clip was considered difficult to interpret. Specifically, trauma patients with subcutaneous emphysema and supine obese patients with significant distance to the lung tissue represent a technical challenge. The division of the chest into six zones as performed in the BLUE protocol and the ICU-SOUND protocol allows rapid assessment of anterior and posterior-lateral fields [15, 18]. Other studies have utilized more lung fields, with the international consensus statement specifying an eight-zone protocol published by Volpicelli et al. [7]. Our study was designed as a rapid diagnostic tool leading to a binary outcome, but the diagnosis of ARDS in other ultrasound studies was one of several potential outcomes at the end of a diagnostic algorithm. By distilling the process to simply an “A-line” or “B-line” determination for each lung field, much of the information we acquired in the process of ultrasound was disregarded, including the presence or absence of lung sliding and images that showed clear signs of consolidation, hepatization or effusions. In the BLUE protocol these findings would have potentially changed the final ultrasound-based diagnosis, and likely contributed to the moderate interobserver agreement we observed. Six points of examination may also not be sufficient for clear identification of alternative processes, as the study that most accurately identified ARDS using pulmonary ultrasound did so by scanning each intercostal space [11].

Alternative methods for ascertainment of imaging and oxygenation criteria for ARDS may be useful in a variety of settings where critically ill patients are managed. Ultrasound evaluation may be faster and offer additional benefits compared to chest radiography [12, 19]. In low-resource settings without portable chest radiography and ABG testing capacity, pulse oximeters and ultrasound machines are increasingly available [3, 4]. Ultrasound is a useful imaging modality in these settings due to its versatility and portability. Moreover, dependence on traditional tools for diagnosing ARDS in low-resource settings may substantially underestimate the incidence of disease [2]. The diagnosis of ARDS is important even when resources are limited because two of the management strategies demonstrated to improve mortality in ARDS – lung protective ventilation and proning [20, 21] – are cheap and potentially feasible to implement in a range of settings. While some evidence supports more liberal use of lung protective ventilation in respiratory failure [22], understanding the prevalence of ARDS is one element in a necessary effort to improve detection and treatment of respiratory diseases and critical illness in low-resource settings globally [23–27].

Our study offers several strengths. First, our study was conducted by a novice sonographer quickly and without moving the patient. While several other studies have examined the ability of ultrasound to identify findings consistent among patients with ARDS [10–12, 18], conditions were optimized: patients were positioned carefully and expert sonographers obtained multiple ultrasound findings in combination. Thus our study was pragmatic and modeled “real-world” conditions for busy ICU practitioners. Second, we methodically evaluated a sequentially enrolled cohort of critically ill medical and surgical patients at risk for ARDS in a large referral center, suggesting external validity of our study to other busy critical care centers. Third, we enrolled patients with a range of diseases and PaO_2_/FiO_2_ ratios; thus our tests of diagnostic accuracy measures should apply to similar spectra of disease. Fourth, we carefully considered the timing of various diagnostic tests in relation to each study observation in order to minimize effects of temporal changes in clinical condition on the analysis. Fifth, although we treated each assessment as independent, we confirmed that intraindividual correlation did not alter our findings. Finally, we tested interobserver effects by performing a secondary analysis of ultrasound characteristics using an independent ultrasound-trained physician to classify images.

Our study also has several limitations. Most notably, this was a small, single-center study with ultrasound data obtained by only one sonographer. Furthermore, few patients had SpO_2_ < 97 %, limiting the number of observations that could be analyzed according to the methodology of Rice et al. [5]. As this was an observational study, additional patients were excluded from analysis if the duration of time between their radiograph or ABG and the study observation was too long. These restrictions resulted in a small population for final analyses. In addition, if less ill patients had less frequent diagnostic tests, our analyses may have been biased towards sicker patients. While the study physician was blinded to the clinical picture for initial assessments, repeat assessments were performed after medical record review, potentially leading to bias. Evaluating only intubated patients limits the generalizability of our results, particularly to lower resourced settings. As noted above, a six-point protocol may be insufficient and a binary “B-line predominant” determination may result in disregarding potentially important clinical information. Avoiding patient repositioning meant that there was limited visualization of the posterior lung fields, which is particularly relevant as ARDS is a posterior predominant condition. Potentially, diagnostic yield would have been higher had assistance been sought. Furthermore, chest radiography is a suboptimal gold standard when compared to chest computed tomography imaging [12].

## Conclusions

The combination of pulse oximetry and six-point rapid bedside pulmonary ultrasound assessment provides a reasonably sensitive method for identifying intubated patients who meet standard ARDS oxygenation and imaging criteria. Future, larger studies in high- and low-resource settings are needed to validate and refine the utility of these modalities in diagnosing lung disease in critically ill patients in under-resourced settings.

## Key messages

- In this pilot study of ICU patients receiving invasive mechanical ventilation, a simplified six-point pulmonary ultrasound assessment for “B lines” is sensitive in identifying bilateral opacities on chest radiography consistent with ARDS.
- Pulse oximetry-derived SpO_2_/FiO_2_ ≤ 315 is a sensitive threshold for PaO_2_/FiO_2_ ≤ 300; pulse oximetry-derived SpO_2_/FiO_2_ ≤ 235 is a specific threshold for PaO_2_/FiO_2_ ≤ 200.
- The combination of pulmonary ultrasound assessment and SpO_2_/FiO_2_ measurement provides a reasonably sensitive method to identify patients who meet conventional ARDS oxygenation and imaging criteria.
- Based on this pilot investigation, further studies to quantify the performance of alternative ultrasound protocols to identify conditions such as ARDS are warranted.

## Additional files

Additional file 1: Figure S1. Receiver operating curves for SpO_2_/FiO_2_ ratio in discriminating PaO_2_/FiO_2_ ≤ 300 (A) and in discriminating PaO_2_/FiO_2_ ≤ 200 (B) when SpO_2_ ≤ 97 %. Additional file 2: Figure S2. Receiver operating curve for UIS pattern in discriminating bilateral opacities consistent with ARDS on chest radiograph. Three thresholds for UIS were defined: B lines in at least one lung field bilaterally, in at least one field bilaterally and involving a minimum of three lung fields, or in at least two lung fields bilaterally.

## Abbreviations

ABG: Arterial blood gas
ARDS: Acute respiratory distress syndrome
AUC: Area under the curve
CI: Confidence interval
CPAP: Continuous positive airway pressure
FiO_2_: Fraction of inspired oxygen
IQR: Interquartile range
PaO_2_: Partial pressure of oxygen
PEEP: Positive end-expiratory pressure
SpO_2_: Pulse oximetric oxygen saturation
UIS: Ultrasound interstitial syndrome

## References

[1] Bernard GR, Artigas A, Brigham KL, Carlet J, Falke K, Hudson L (1994). The American-European Consensus Conference on ARDS. Definitions, mechanisms, relevant outcomes, and clinical trial coordination. Am J Respir Crit Care Med.

[2] Riviello E, Talmor D, Novack V, Kiviri W, Twagirumugabe T, Banner-Goodspeed V (2014). Incidence and outcomes of ARDS in Rwanda using modified Berlin criteria for resource-poor settings. Crit Care Med.

[3] Sippel S, Muruganandan K, Levine A, Shah S (2011). Review article: use of ultrasound in the developing world. Int J Emerg Med.

[4] Funk LM, Weiser TG, Berry WR, Lipsitz SR, Merry AF, Enright AC (2010). Global operating theatre distribution and pulse oximetry supply: an estimation from reported data. Lancet.

[5] Rice TW, Wheeler AP, Bernard GR, Hayden DL, Schoenfeld DA, Ware LB (2007). Comparison of the SpO2/FIO2 ratio and the PaO2/FIO2 ratio in patients with acute lung injury or ARDS. Chest.

[6] Khemani RG, Patel NR, Bart RD, Newth CJ (2009). Comparison of the pulse oximetric saturation/fraction of inspired oxygen ratio and the PaO2/fraction of inspired oxygen ratio in children. Chest.

[7] Volpicelli G, Elbarbary M, Blaivas M, Lichtenstein DA, Mathis G, Kirkpatrick AW (2012). International evidence-based recommendations for point-of-care lung ultrasound. Intensive Care Med.

[8] Lichtenstein D, Meziere G, Biderman P, Gepner A (1997). The comet-tail artifact. An ultrasound sign of alveolar-interstitial syndrome. Am J Respir Crit Care Med.

[9] Volpicelli G, Mussa A, Garofalo G, Cardinale L, Casoli G, Perotto F (2006). Bedside lung ultrasound in the assessment of alveolar-interstitial syndrome. Am J Emerg Med.

[10] Lichtenstein DA, Meziere GA (2008). Relevance of lung ultrasound in the diagnosis of acute respiratory failure: the BLUE protocol. Chest.

[11] Copetti R, Soldati G, Copetti P (2008). Chest sonography: a useful tool to differentiate acute cardiogenic pulmonary edema from acute respiratory distress syndrome. Cardiovasc Ultrasound.

[12] Lichtenstein D, Goldstein I, Mourgeon E, Cluzel P, Grenier P, Rouby JJ (2004). Comparative diagnostic performances of auscultation, chest radiography, and lung ultrasonography in acute respiratory distress syndrome. Anesthesiology.

[13] Bedetti G, Gargani L, Corbisiero A, Frassi F, Poggianti E, Mottola G (2006). Evaluation of ultrasound lung comets by hand-held echocardiography. Cardiovasc Ultrasound.

[14] Baker K, Mitchell G, Stieler G (2013). Limited lung ultrasound protocol in elderly patients with breathlessness; agreement between bedside interpretation and stored images as acquired by experienced and inexperienced sonologists. Australasian J Ultrasound Med.

[15] Lichtenstein DA, Mezière GA (2011). The BLUE-points: three standardized points used in the BLUE-protocol for ultrasound assessment of the lung in acute respiratory failure. Critical Ultrasound Journal.

[16] Lichtenstein DA (2014). Lung ultrasound in the critically ill. Ann Intensive Care.

[17] Ranieri VM, Rubenfeld GD, Thompson BT, Ferguson ND, Caldwell E, Fan E (2012). Acute respiratory distress syndrome: the Berlin Definition. JAMA.

[18] Manno E, Navarra M, Faccio L, Motevallian M, Bertolaccini L, Mfochive A (2012). Deep impact of ultrasound in the intensive care unit: the “ICU-sound” protocol. Anesthesiology.

[19] Ashton-Cleary DT (2013). Is thoracic ultrasound a viable alternative to conventional imaging in the critical care setting?. Br J Anaesth.

[20] Determann RM, Royakkers A, Wolthuis EK, Vlaar AP, Choi G, Paulus F (2000). Ventilation with lower tidal volumes as compared with traditional tidal volumes for acute lung injury and the acute respiratory distress syndrome. The Acute Respiratory Distress Syndrome Network. N Engl J Med.

[21] Guerin C, Reignier J, Richard JC, Beuret P, Gacouin A, Boulain T (2013). Prone positioning in severe acute respiratory distress syndrome. N Engl J Med.

[22] Serpa Neto A, Cardoso SO, Manetta JA, Pereira VG, Esposito DC, Pasqualucci Mde O (2012). Association between use of lung-protective ventilation with lower tidal volumes and clinical outcomes among patients without acute respiratory distress syndrome: a meta-analysis. JAMA.

[23] Einav S, Hick JL, Hanfling D, Erstad BL, Toner ES, Branson RD (2014). Surge capacity logistics: care of the critically ill and injured during pandemics and disasters: CHEST Consensus Statement. Chest.

[24] Hick JL, O’Laughlin DT (2006). Concept of operations for triage of mechanical ventilation in an epidemic. Acad Emerg Med.

[25] Adhikari NK, Fowler RA, Bhagwanjee S, Rubenfeld GD (2010). Critical care and the global burden of critical illness in adults. Lancet.

[26] Dunser MW, Baelani I, Ganbold L (2006). A review and analysis of intensive care medicine in the least developed countries. Crit Care Med.

[27] Biddison LD (2014). Ethical considerations: care of the critically ill and injured during pandemics and disasters: CHEST consensus statement. Chest J.

