# Supplementary figures and images for: Pulmonary ultrasound and pulse oximetry versus chest radiography and arterial blood gas analysis for the diagnosis of acute respiratory distress syndrome: a pilot study
Source: Crit Care. 2015 Jul 21;19(1):282. doi: 10.1186/s13054-015-0995-5 (PMC4511255; doi:10.1186/s13054-015-0995-5)

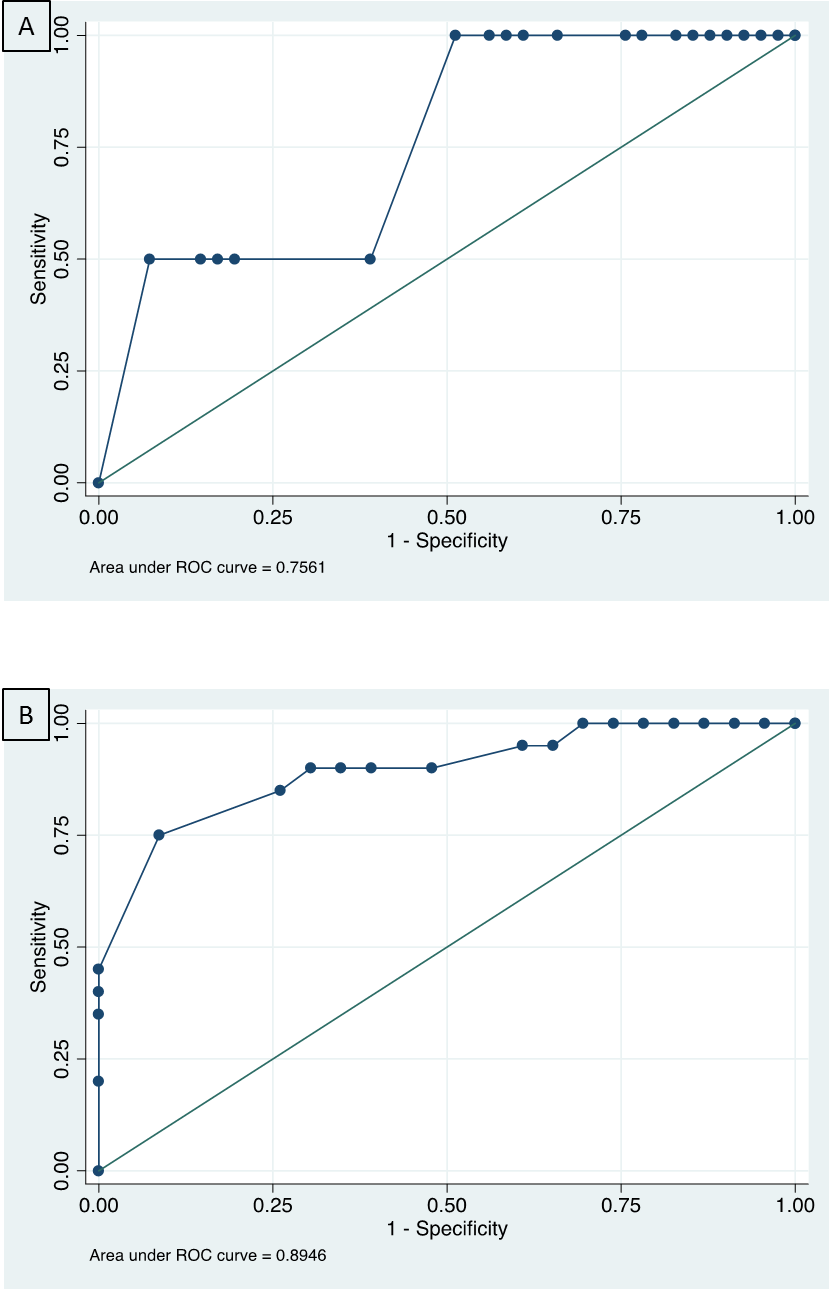

Supplement: Additional file 1: Figure S1. — Receiver operating curves for SpO2/FiO2 ratio in discriminating PaO2/FiO2 ≤ 300 (A) and in discriminating PaO2/FiO2 ≤ 200 (B) when SpO2 ≤ 97 %. [file 13054_2015_995_MOESM1_ESM.tif]

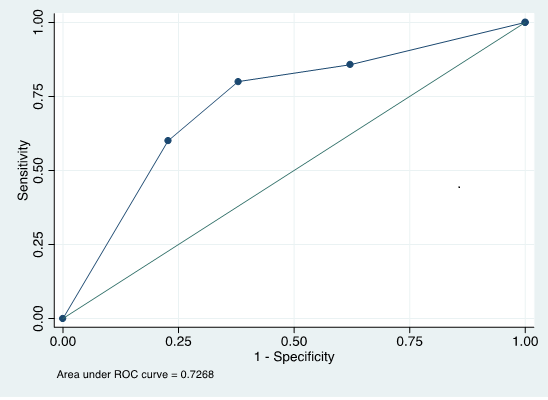

Supplement: Additional file 2: Figure S2. — Receiver operating curve for UIS pattern in discriminating bilateral opacities consistent with ARDS on chest radiograph. Three thresholds for UIS were defined: B lines in at least one lung field bilaterally, in at least one field bilaterally and involving a minimum of three lung fields, or in at least two lung fields bilaterally. [file 13054_2015_995_MOESM2_ESM.tif]
